# Does aroma influence Indigenous and local people’s selection and utilization of plants? A scoping review

**DOI:** 10.1186/s13002-026-00930-4

**Published:** 2026-07-17

**Authors:** Kimberly P. Castro, Rodrigo Cámara-Leret

**Affiliations:** Department of Systematic and Evolutionary Botany, University of Zurich, Zurich, Switzerland

**Keywords:** Food plants, Human–plant interactions, Organoleptic properties, Plant aroma, Smell perception, Taste perception, Volatile organic compounds

## Abstract

**Background:**

The influence of morphological traits on the selection and utilization of plants has been widely investigated, but explaining how people recognize a plant’s usefulness based on chemistry has been challenging. Plant aroma, as a perceptual indicator of underlying chemical composition, may play an important role in this process, yet its contribution remains insufficiently understood.

**Methods:**

We conducted a review of literature published over the past 50 years to examine the role of plant aroma and human olfaction in shaping plant selection and perceived utility within Indigenous and local knowledge systems. We assessed patterns of interdisciplinarity by combining qualitative content and co-occurrence analyses with matrix-based heatmap visualizations.

**Results:**

We find that aroma contributes, to some extent, to the selection and utilization of plants by facilitating preliminary identification, therapeutic classification, and intergenerational knowledge transmission. However, the mechanisms underlying this relationship remain poorly understood mainly due to the lack of interdisciplinary approaches and methodological limitations in most studies.

**Conclusions:**

Limited integration across disciplines constrains definitive conclusions, underscoring the need for more rigorous interdisciplinary research. We propose integrating phytochemistry with standard ethnobotanical data collection to better understand the role of plant aroma and olfactory perception in plant selection.

**Supplementary Information:**

The online version contains supplementary material available at https://doi.org/10.1186/s13002-026-00930-4.

## Introduction

Humans have long relied on plants for food and medicine, and understanding how plants are selected for these uses has been a central concern of ethnobotany [79]. Contemporary ethnobotanical research has moved beyond descriptive inventories toward examining the social, ecological, and perceptual factors that shape plant use. Studies on human–plant interactions have highlighted the importance of morphological traits, cultural transmission and experiential learning in plant selection and utilization [8, 12, 83]. Alongside these factors, plant chemical traits have been associated with patterns of use, particularly in relation to food and medicine [42, 72, 86]. However, the role of plant chemistry as it is *perceived* by humans—rather than as analytically defined—has received comparatively less attention since it is not directly accessible, although it is often inferred through sensory cues such as smell, taste, and other organoleptic properties that guide recognition, valuation, and use of plants.

**Fig. 1:**
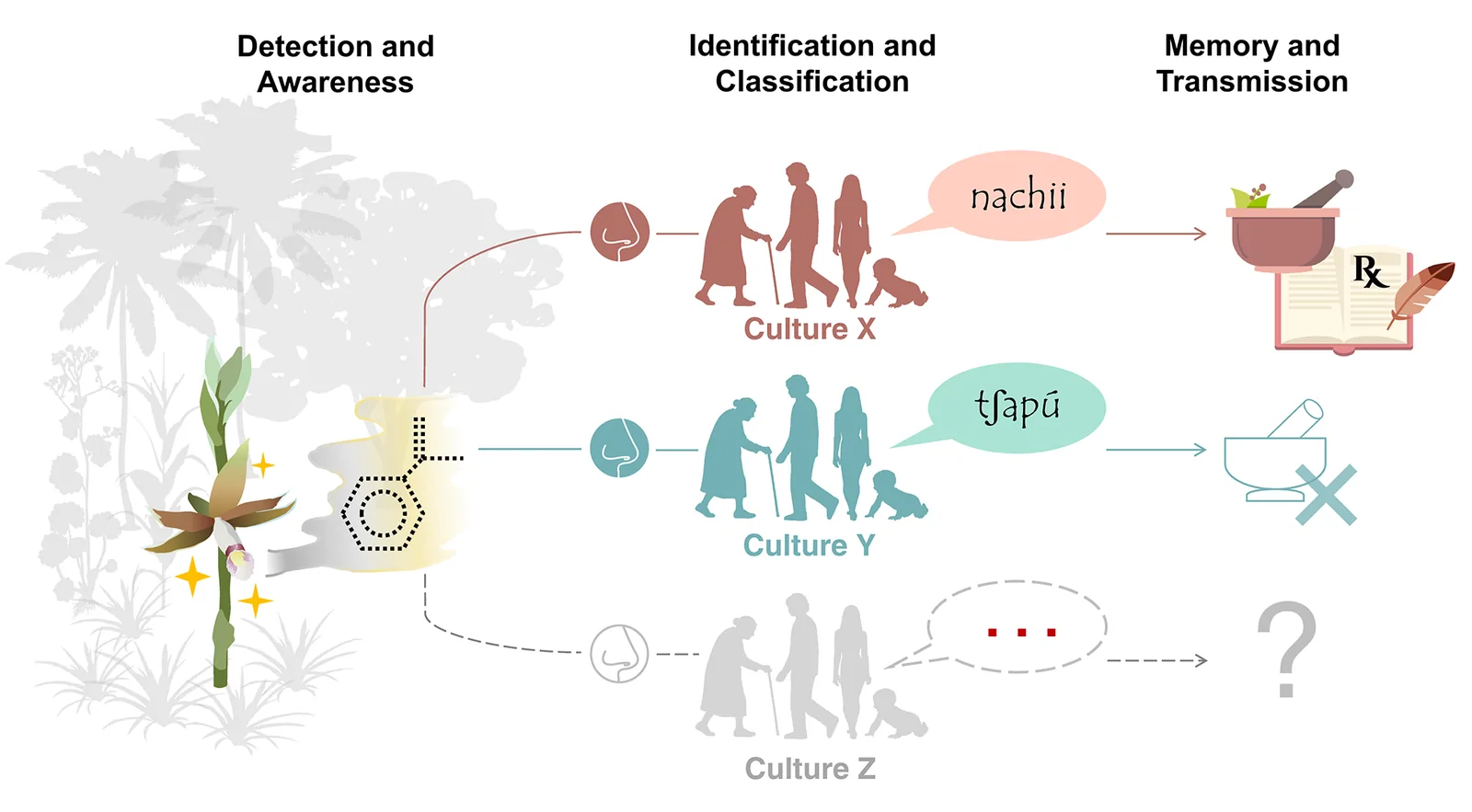
Conceptual framework illustrating how plant aroma may influence human-plant interactions. Volatile organic compounds emitted by plants are detected and perceived through olfaction, after which they are identified, classified, and communicated using culturally specific perceptual systems (represented by color coded community silhouettes) and odor vocabularies (e.g., *nachii* can be roughly translated to “rich odor” in Tikuna language, while *tʃapú* can be roughly translated to “harm” in Yucuna language). These classifications may influence decisions regarding plant selection and utilization and contribute to the transmission of Indigenous and local knowledge. Differences in cultural interpretation and odor lexicons can result in variation in how the same plant aroma is perceived, categorized, and acted upon across societies.

The phytochemical properties of many medicinal plants have been extensively investigated in pharmacognostic and pharmacological research. In contrast, far fewer ethnobotanical studies have explicitly examined how such properties are perceived, interpreted, and acted upon through sensory experience, particularly smell. Influential frameworks to discuss human–plant interactions, such as ecological and coevolutionary hypotheses, were largely derived by interactions between plants and non-human organisms and do not directly address human perceptual processes [28, 42]. Moreover, application of these frameworks in ethnobotany is often uncritical, relying on simplified proxies and leading to ambiguous or potentially misleading conclusions [52]. Because these approaches rarely account for the interplay between plant chemistry, ethnobotany, and human sensory experience, they contribute to the continued compartmentalization of insights across disciplines. This fragmentation limits our understanding of how aroma functions as a mediating link between plant chemical traits and human plant selection, particularly within Indigenous and local knowledge systems where sensory experience plays a central role (Fig. 1).

Plants produce a vast diversity of specialized metabolites, a subset of which—volatile organic compounds (VOCs)—contribute substantially to aroma and play key roles in ecological interactions by mediating communication between plants and other organisms [22, 27, 86]. For example, specific VOCs (e.g., 1,8-cineole, benzyl acetate, α-pinene) in the scent of orchid flowers attract pollinators like euglossine bees, while aromatic cues in other higher animals like lemurs can inform foraging decisions by signaling fruit ripeness [19, 66, 78]. While these studies illustrate general principles of aroma-mediated plant–animal interactions, their direct applicability to human perception is limited due to differences in sensory physiology, cognition, and cultural interpretation.

In this review, we define ‘plant aroma’ as the perceptual experience triggered by VOCs released from plant tissues. We emphasize that aroma cannot be reduced to chemical composition alone, but emerges from interactions among VOCs, human olfactory physiology, cognitive processing, and cultural context. Accordingly, an “aroma profile” is understood here as a perceptual and cultural construct rather than a direct proxy for a phytochemical fingerprint. Despite olfactory perception being transient, subjective, and difficult to observe directly [16], linguistic descriptions of smell play a crucial role as an accessible empirical trace for capturing, structuring, and transmitting olfactory knowledge within and across cultures [56]. Odor vocabularies, metaphors, and classificatory systems therefore constitute critical evidence for understanding how people conceptualize plant aromas and associate them with specific uses. Integrating linguistic evidence with ethnobotanical observations and sensory research could thus enable a more nuanced examination of aroma-mediated human–plant interactions than any single disciplinary approach.

Here, we present a conceptual and integrative ethnobiological review synthesizing literature published over the past 50 years on the relationship between plant aroma and human plant selection and use. Specifically, we (1) examine how plant aroma and olfaction have been investigated across ethnobotanical, sensory, and chemical studies; (2) assess the role of cultural and linguistic diversity in shaping the perception and communication of plant aromas; and (3) identify conceptual and methodological gaps that constrain interdisciplinary integration. Rather than providing a definitive answer to whether plant aroma influences selection and utilization of plants, this review evaluates the extent to which existing evidence from ethnobotany, phytochemistry, sensory science, and linguistic studies addresses this question. By identifying recurring patterns and persistent limitations across these disciplines, we highlight opportunities for interdisciplinary research on how plant chemical properties are recognized and acted upon within Indigenous and local knowledge systems.

## Materials and methods

### Search strategy

We conducted a literature review following the PRISMA 2020 guidelines [69]. A comprehensive literature search was performed in the Scopus database in January 2024. Scopus was selected as it provides broad coverage of peer-reviewed literature in ethnobotany and includes both life sciences and social sciences indexed journals. The search string was designed to capture studies at the intersection of ethnobotany, olfactory perception, and chemistry. We combined terms related to plants and traditional knowledge (ethno*, “medicinal plant”) and sensory properties (organoleptic, sensor*, aroma*, scent*, taste*, olfaction, fragran*, flavo?r*, odo?r*) using Boolean operators (AND, OR). Wildcards (*) were used to capture word variations, and spelling variants were included (e.g., odo?r* = odor/odour). Database-specific filters were applied to limit results to articles and reviews. We restricted the search to publications in English, Spanish, and Portuguese due to language proficiency constraints. The search was limited to publications from 1970 to 2023. The initial database search retrieved 9,813 documents.

Citation tracking was performed to identify additional relevant studies. Reference lists of included articles were manually screened for potentially relevant publications not captured in the database search (backward citation tracking). Additionally, we tried to identify more recent publications that cited the included studies (forward citation tracking). Studies identified through citation tracking were screened using the same inclusion and exclusion criteria.

### Screening and selection

A two-stage screening process was employed. First, titles and abstracts were screened manually using predefined inclusion and exclusion criteria. The majority of records were readily excluded based on title alone as they obviously addressed unrelated topics such as synthetic chemistry, food and flavor science, non-human sensory interactions, or studies that were not plant-focused. Articles containing potentially relevant terms were further assessed for abstract screening. Studies were included if they met all of the following criteria: (1) investigated plants, (2) focused on Indigenous people or communities with traditional/local knowledge, (3) mentioned smell/aroma/chemosensory property and substantially discuss its role in plant selection, identification, quality assessment, or utilization by the studied communities. Taste is considered only where it overlaps with retronasal olfaction in aroma perception, and the primary focus remains on aroma as perceived through smell. No automated text-mining or machine-learning screening tools were used. Those that were classified as potentially relevant after title and abstract screening (*n* = 51) advanced to full-text review. Full-text articles were evaluated against all inclusion criteria, and studies meeting all criteria (*n* = 29) were included in the final analysis. The selection process is illustrated in Figure S1.

### Data extraction

For each included study, the following information was systematically recorded: publication year, biogeographic region, Indigenous culture or local community studied, data collection methods (interviews with people, database, journal articles), study focus (medicinal plants, food plants), plant types (wild, cultivated, or both), total number of plant species reported, inclusion of sensory evaluation methods, documentation of local odor terminologies or classification systems, discussion of relationships between sensory properties and phytochemistry, inclusion of phytochemical analyses, and challenges or limitations and recommendations recognized by the authors. The complete extracted data for all included studies are provided in Supplementary Table S1.

### Data analyses

To examine the degree of conceptual integration of the literature, we analyzed commonly used terms through a co-occurrence analysis using the VOS Viewer software (version 1.6.20) [23]. This analysis is an exploratory bibliometric approach to identify dominant research foci and the extent to which different domains are conceptually interconnected or remain isolated. The analysis was based on all document titles and abstracts from the initial search (*n* = 9,813) extracted through the export function of Scopus database. We used a binary counting method with a 10-occurrence threshold and selected the top 60% frequent terms for mapping, as recommended by the software and by our exploratory analyses of the metadata. The resulting network map groups terms into clusters based on their co-occurrence patterns, which can be interpreted as reflecting underlying thematic or disciplinary structures within the literature.

To characterize the interdisciplinary scope of the included studies in this review, we conducted a structured qualitative content analysis of all retained articles (*n* = 29). Each study was coded according to the presence or absence of predefined analytical dimensions relevant to plant aroma and human plant use. These dimensions were defined a priori based on the objectives of the review and included: (1) explicit consideration of olfactory perception, (2) inclusion of taste or aroma-related information, (3) linguistic or descriptive studies of odor vocabularies, and (4) phytochemical characterization, particularly of VOCs. Each study was coded in a binary manner (presence = 1; absence = 0) based on whether the respective dimension was explicitly addressed in the title, abstract, methods, or results. Coding was performed manually by the authors. The resulting dataset was organized as a study-by-variable matrix and visualized as heatmap to facilitate comparison across studies. No statistical inference was applied, as the aim was descriptive and conceptual rather than quantitative synthesis.

## Results and discussion

### Limited interdisciplinarity and scant human olfaction research

**Fig. 2:**
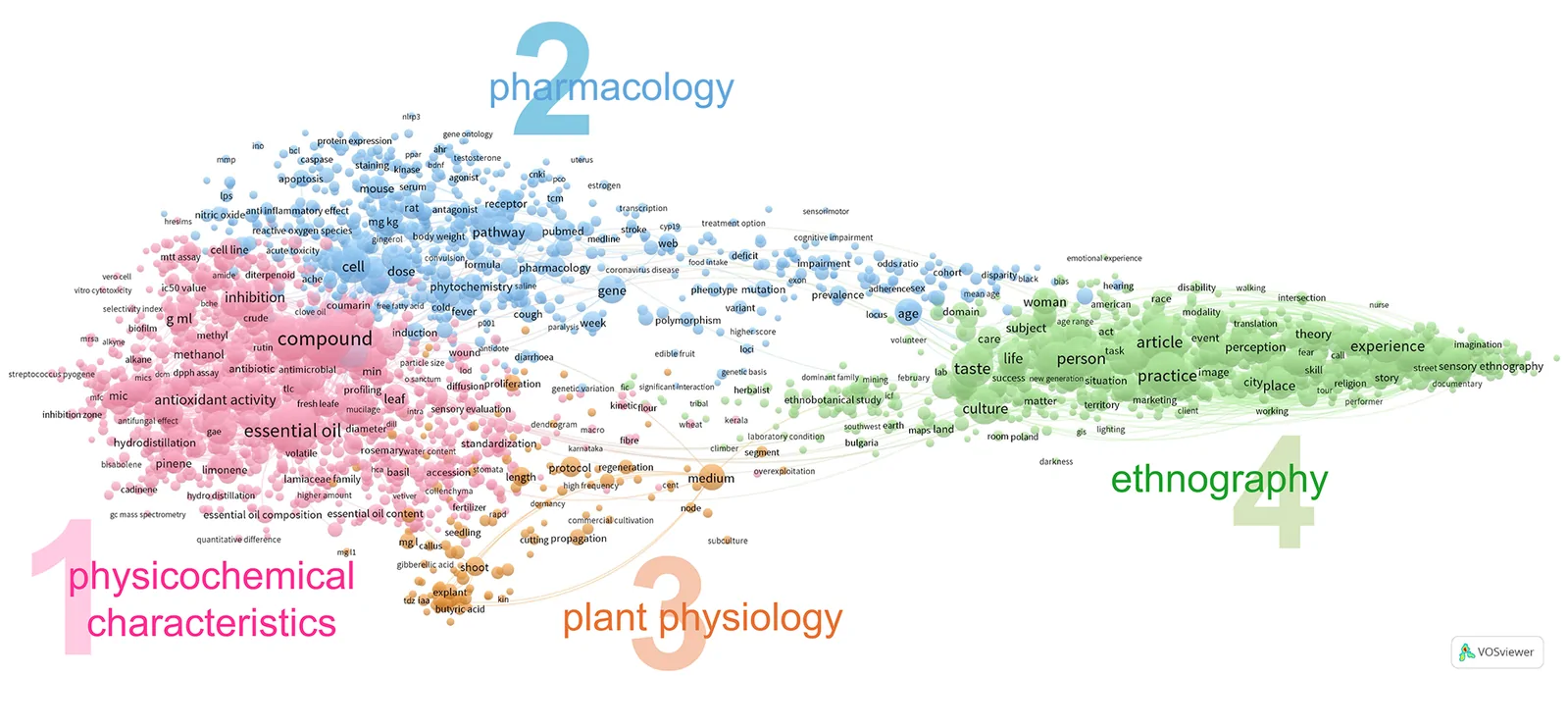
Co-occurrence network of the most frequent terms and their associations from the titles and abstracts of 9,813 publications. Node size is proportional to term frequency in the titles and abstracts. Node color depicts the main clusters formed by the co-occurrence of terms.

From the publications screened (*n* = 9,813), an analysis of term co-occurrence revealed four distinct clusters. Cluster one (Fig. 2 in pink) contains works focusing on the physicochemical characteristics of plants (i.e., extraction, compounds, concentration). Cluster two (Fig. 2 in blue) highlights pharmacological studies, with terms such as activity, mechanism, and pathway. Cluster three (Fig. 2 in orange) shows studies on plant physiology. Lastly, cluster four (Fig. 2 in green) reflects a focus on ethnography, with terms like person, taste, and culture. The strong clustering within disciplinary boundaries, the proximity of clusters 1–3, and isolation of cluster four—that emphasizes the social sciences—evidences the lack of studies bridging natural and social sciences and a restricted focus on single disciplines (i.e., chemistry, pharmacology, physiology, ethnography). Taken together, these co-occurrence patterns indicate that interdisciplinary integration remains limited in studies linking aromas with human plant use.

**Fig. 3:**
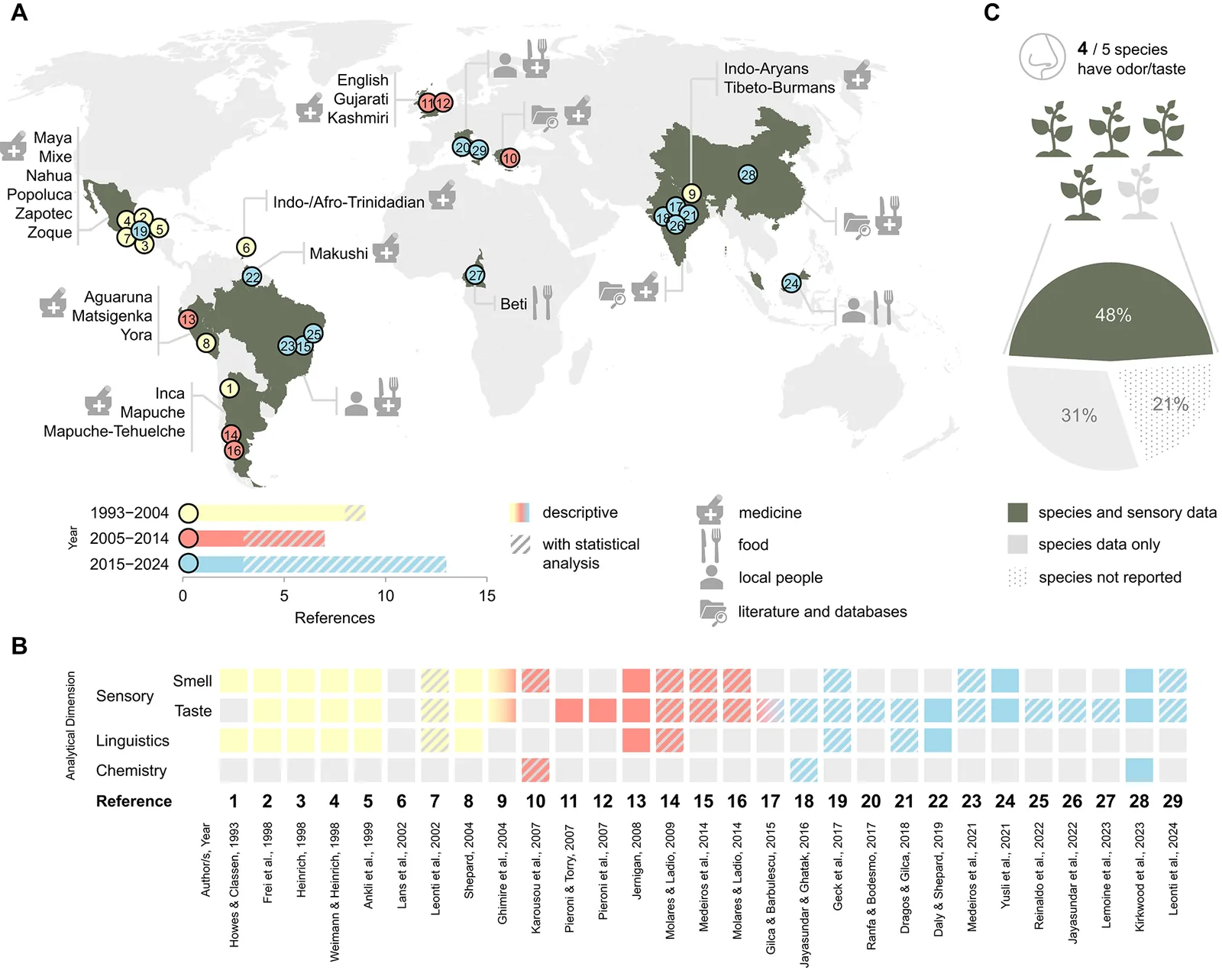
Overview of the current state of research linking plant aroma and use. (**A**) Spatiotemporal trends for 29 references (circles) reviewed in this study. The source of information (Indigenous group, local people, or literature and databases) and the study focus (medicinal or food plants) are specified for each country. Bar plot shows the temporal distribution of references in three periods. (**B**) Degree of interdisciplinarity of the 29 studies included in this review, mapped across three analytical dimensions. Rows represent the dimensions covered in this review (i.e., sensory evaluation, linguistics, and phytochemical analysis) and columns represent studies (sorted by publication year). A cell is filled when that dimension was explicitly addressed in a study. The matrix highlights the fragmented nature of existing research, with 92% of the studies focusing on individual dimensions in isolation and relatively few integrating olfactory perception with chemical and cultural approaches. (**C**) Proportion of studies that reported the total number of species investigated and those with odor and/or taste characteristics.

From the 9,813 studies obtained (including those found through manual search and citation tracking), only 29 references met our search criteria and were analyzed qualitatively (Fig. 3A). This reduction is mainly due to the lack of interdisciplinary research across the natural and social sciences (Fig. 3B). The low number of 29 references for human–plant interaction studies is in stark contrast with the extensive research on chemosensory perception in plant-animal interactions [80] and highlights the considerable potential for interdisciplinary studies that combine experimental and cross-cultural approaches.

### How does aroma influence plant selection and utilization?

Plant aroma and human olfaction contribute to a wide range of activities relevant to plant use, including foraging, food evaluation, and medicinal plant identification [32, 49, 87]. The well-documented continuum between food and medicine further highlights the importance of sensory perception in plant classification and selection, as many species occupy overlapping nutritional and therapeutic roles informed by local knowledge and experience [24, 61, 73]. Among sensory cues, plant aroma occupies a particularly salient yet understudied role in human plant selection and use. Here, we identified three general insights on how human olfaction and plant aromas influence plant selection and utilization.

#### A. Species identification and therapeutic classification

The first general insight emerging from the literature is that plant aromas can complement the visual identification and classification of plants. Even for studies that focused only on a few culturally important plants, they have often found that aroma properties aid in distinguishing between plant species and varieties [30, 34, 50]. For example, mint species in Greece can be classified into four groups based on smell [44]: Group A which are scentless (*Clinopodium alpinum* (L.) Kuntze); Group B with a pungent smell (e.g., *Clinopodium suaveolens* (Sm.) Kuntze, *Mentha pulegium* L., and *Mentha* × *piperita* L.); Group C with a musty smell (*Mentha spicata* L. and *Mentha suaveolens* Ehrh.); and Group D with a sweet smell (*Mentha longifolia* (L.) L. and *Mentha* × *villoso-nervata* Opiz.). Similarly, the Aguaruna people of the Peruvian Amazon assess relatedness of trees based on the odor of the bark, sap, flowers, fruits, and leaves [41].

Studies in the Neotropical region (Fig. 3A) predominantly focused on medicinal plants and aimed to identify correlations between plant organoleptic properties and their therapeutic classifications. Ethnobiological studies have repeatedly noted smell as a salient criterion in the recognition and use of plants, particularly in medicinal contexts [35, 64]. The smell and taste of a plant was found in some studies to predicate its humoral quality (i.e., hot-cold property), which in turn acted as a cultural filter to determine the plant’s therapeutic use [29, 51]. Some use patterns emerged in neighboring Indigenous groups in Central America (Yucatec Maya, Zapotec, Zoque, Mixe, and Popoluca) (e.g., astringent plants treat diarrhea and dysentery, bitter plants supplement astringent plants, sweet/sour plants for respiratory problems, aromatic plants for pain and external ailments) [3, 25, 29, 35, 51]. It is also interesting that some Indigenous groups (e.g., Popoluca of Mexico, Mapuche of Argentina) express a willingness to select substitute plants that have similar organoleptic properties than the original medicinal plant [51, 63]. Similarly, early Western naturalists such as Linnaeus sought to systematize medicinal plant knowledge using smell and taste, as classificatory tools [75, 84]. In line with this, a recent study demonstrated associations between the taste properties of plant-derived compounds and their pharmacological activities reported in Dioscorides’ *De Materia Medica*, suggesting that human perception may provide meaningful cues about bioactivity [53]. However, because the perception and interpretation of chemosensory stimuli are strongly shaped by culture [5, 10, 74] any generalizations on therapeutic classifications based on organoleptic properties will require understanding the perceptions of far more societies than the single one typically covered in individual studies. Inclusion of diverse linguistic and ecological contexts will be crucial, as our ability to describe odors relies on—and is embedded within—this prior experience, or the “happenstance of individual lives” [59].

#### B. Detection and awareness

A second insight in the literature is that having a characteristic aroma makes a plant more apparent and therefore increases its likelihood to be perceived, selected, and used as a medicine (i.e., sensory apparency [82]). This is analogous to findings from plant-animal interaction research, where VOCs attract or deter pollinators and herbivores. However, unlike the clear role of VOCs in plant-animal interactions, human association of distinct odors or taste with medicinal plant properties remains poorly understood and rarely tested [7, 18]. In studies reporting plant species counts (Supplementary Table S1), about 4 in 5 species have odor or taste characteristics (Fig. 3C), suggesting that aromatic plants dominate local pharmacopoeia. However, this pattern is not straightforward, for at least three reasons. First, informants were usually asked to select medicinal or useful plants. Second, little is still known about the organoleptic properties of the remaining “non-medicinal” flora, and no study has yet investigated the regional species pool (medicinal and non-medicinal) that informants are exposed to. One of the only two reviewed studies in which healers were asked to also select plants with no medical value reported that “non-medicinal” plants (17% species studied) were inconspicuous (i.e., fruits and flowers did not smell or taste) [3]. Finally, most studies (72%) do not explicitly state whether the investigated plants are wild or cultivated (5 studies considered both, 2 studies focus on cultivated plants, and only 1 studied wild plants). These limitations make it difficult to assess whether plant selection is driven by distinct organoleptic properties in medicinal vs. non-medicinal plants and/or how this applies to wild, non-cultivated plants.

#### C. Memory and knowledge transmission

The third trend identified in the literature is that the perceived smell and taste of plants can function as a mnemonic aid [7, 28, 51]. Just as in plant-animal interactions where a repeated exposure of pollinators to certain VOCs enables learning about a “reward” [9], the possibility arises that humans may use the perceived smell or taste of a plant to infer its efficacy, reinforcing its medicinal value within a culture [25, 74]. Some studies suggest that the preference for bitter-tasting medicinal plants may arise from cultural learning, as it contrasts with the general avoidance of bitterness due to toxicity [11, 60]. Eventually, the regular use of a plant solidifies its importance in a culture; and thus, becomes a part of knowledge passed down through generations [88]. Attaching a tangible sensation and additional descriptor to plants may facilitate remembering and disseminating their identity and use, especially among non-literate societies. As an example, the Mapuche—through their language *Mapudungun*—have richer organoleptic descriptions for the most frequently used plant species [63], as do local people in Northeastern Brazil [60].

The impact of plant aroma and olfaction on intergenerational knowledge transmission is also reflected in the changing trend in information gathered over time by the researchers. On the one hand, early studies from the Neotropics were descriptive and investigated how Indigenous and local people describe plant species’ smell and taste (Fig. 3B). On the other hand, studies in the last decade increased in complexity and integrate ecological and cultural correlates of a plant’s importance with organoleptic properties. For example, recent works ask whether people *prefer* the smell or taste of the plant in combination with other explanatory factors such as plant availability or versatility. Accordingly, more sophisticated analyses are employed such as multivariate techniques (e.g., analysis of variance, principal component analysis), non-parametric tests (e.g., Chi-square test, Kruskal-Wallis test, Spearman’s rank correlation) or stochastic modelling (e.g., Bayesian statistics, Monte Carlo simulations). Meanwhile, recent studies in Asia did not directly involve people but drew on literature [48], databases [21, 31], or paired phytochemical analyses with technologies like taste sensing systems [39] to relate chemosensory perception with human–plant interaction (Fig. 3A). Nevertheless, direct sensory testing remains important, as database-derived associations between odor or taste and plant use may not adequately capture perceptual complexity [31]. In summary, there is a trend in methodological refinement in how plant aromas are studied in relation to plant selection, with more recent studies addressing the links of aromas to cultural importance and advocating for more analytical work.

### To what degree does aroma influence plant selection and utilization?

**Fig. 4:**
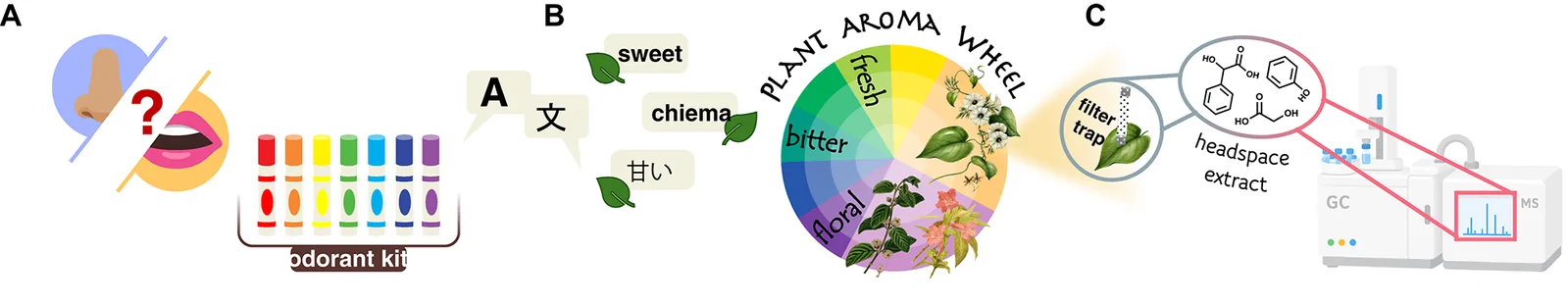
Proposed recommendations to address key research gaps through interdisciplinary approaches: (**A**) establishing standardized frameworks for odor and taste perception and data collection; (**B**) incorporating Indigenous and local linguistic dimensions (e.g., development of an aroma wheel); and (**C**) characterizing plant aroma through volatilomics.

Below, we discuss three key research gaps that emerged from our literature review and that collectively limit our understanding of the influence of aroma in plant selection and utilization (Fig. 4). Addressing these gaps is essential for three reasons: (1) to develop a robust framework linking plant chemistry, human sensory perception, and cultural knowledge systems; (2) to advance methodological integration across ethnobotany, sensory science, linguistics, and phytochemistry; and (3) to enable precise investigation of a more nuanced question: *to what degree does aroma influence plant selection and utilization?*

#### A. Understanding the differences between and within senses

The idea that humans select and utilize plants based on smell and/or taste implies that these sensory qualities are structurally perceived and categorized. These classifications, in turn, guide plant selection and utilization (e.g., plants with particular sensory profiles are associated with specific purposes). Chemosensory perception, particularly olfaction, begins when VOCs bind to olfactory receptors in the nose and commence a signaling cascade that is eventually decoded in the higher cortical regions of the brain [57, 65]. VOCs and olfaction are likewise involved in perceiving “flavor”—the combined sensation of smell, taste, and touch—because most flavors arise when VOCs from the mouth are forced into the nose [14, 32]. Hence, smell and taste are tightly intertwined (i.e., flavors) and often interpreted together, complicating the identification of distinct quality attributes and the unambiguous association of plants with these attributes [64, 74]. For example, although *Alvaradoa amorphoides* Liebm. is not very fragrant, the Yucatec Maya describe it as strong smelling probably because of its strong and very characteristic taste [3]. The same goes for the ethnopharmacological classification used in Ayurvedic medicine called *rasa* which—although roughly translates to taste—includes all sensations of taste, smell, and even touch [21, 31]. On the other hand, the Japanese classification of agarwood (*Aquilaria* Lam.) is also based on so-called “flavors” but mostly pertains to scent [17].

Within senses there also comes a variability in terms of quality attributes. In the reviewed literature, quality attributes for smell ranged from two to 12, while those for taste ranged from four to 10. The human sense of taste has five basic qualities that are consistent among individuals across cultures, namely: sweet, bitter, salty, sour, and umami [6, 14]. This may explain why most references included taste and found a greater consensus on taste qualities than smell. Identifying basic qualities for the human sense of smell, on the other hand, can be challenging. Although olfactory perception starts when VOCs bind to olfactory receptors, VOC and olfactory receptor stimulation is not a one-to-one correspondence. It operates on a combinatorial coding scheme resulting to a myriad possibilities of perceived odor quality [26, 76, 77]. While this fact might make uncovering all the peculiarities of odor qualities in a culture impossible, some studies show that the inclusion of odorant kits in methodological testing may help identify a higher number of quality attributes (Fig. 4A) (e.g., in [81, 87]). Odorant kits are used in clinical setting to test various olfactory functions such as olfactory identification, olfactory discrimination, and olfactory threshold [20, 37]. Since odorant kits are generally composed of common everyday odors, they can also help in making the sensation relatable and thereby facilitate standardized data collection and scientific communication [81].

#### B. Revisiting the limits of language

Identifying quality attributes is one thing, but communicating or naming them is another, although the two are obviously connected [43]. Since language requires cognition and cognition reflects how humans perceive the environment [35], the inability of humans to name quality attributes—particularly smell—may be one of the reasons olfaction is undervalued and often disregarded. Nevertheless, beliefs that humans have poor olfaction or that smells are ineffable are increasingly being challenged [55, 58]. A growing body of work shows that non-Western languages possess extensive vocabularies to describe a multitude of odors [55]. Hence, one promising approach to study Indigenous and local plant use is through an ethnolinguistic perspective [35]. However, only 12 of the 29 references analyzed in this review studied local odor and taste terms (Fig. 3B). The rest fit the local terms/classification onto the terms/classification known to Western languages (e.g., pleasant, strong, bad). Other studies employ the Likert scale to gauge smell and taste preference. These approaches simplify analyses and facilitate communication, but may have unwarranted consequences, for two reasons. First, sorting local terms to fit Western classification systems may miss specific or even unique quality attributes to a particular non-Western culture [60, 70]. For example, the Quechua language in Peru has 23 terms related to olfaction with nuances between seemingly equivalent terms like *curayani* (“to smell bad”) and *ccuncucay* (“to smell *naturally* bad”) [36]. The Nahuatl language in Mexico uses three different modifiers for the taste quality *chichic* (“bitter”) which can be used in differentiating plant taste characteristics [85]. Second, fitting local terms to Western classification system could also imply imposing the Western perspective of perceiving smells and tastes to an entirely different perceptual system. Jraissati and Deroy, [43] argue that odor classification, like any other senses, is dependent on the local olfactory environment known to a person. This is probably the reason why some smell and taste in a given locality are simply too difficult to express or lack an exact translation to Western language [21, 31, 55]. But locality does not only refer to a place or a culture for it may also pertain to specialized fields like perfumery or wine tasting. Experts in these fields are constantly exposed and trained to recognize and develop the skill to discriminate a set of relevant odors along with corresponding terminologies [43, 59]. To standardize and facilitate communication of odor terminologies among experts and the public, aroma wheels are often used (Fig. 4B). Aroma wheels are useful visual representations illustrating the relationships among quality attributes or specific odor-source qualities [62, 67]. Broad descriptor categories (e.g., floral, fruity, or earthy) are typically positioned in the inner circle, while progressively more specific odor terms are projected outwards the wheel. The categories are also organized according to perceived similarities among odor qualities, providing a logical scheme for describing and comparing aroma perceptions. Creating an aroma wheel to represent a culture’s olfactory space offers promise to effectively communicate people’s perceptions of their surrounding smellscapes through their own language. It may provide a useful framework for documenting and comparing culturally specific odor vocabularies while maintaining links to sensory and chemical characteristics. To the best of our knowledge, the use of Indigenous aroma wheels to transmit and preserve traditional knowledge remains unexplored.

#### C. Leveraging the era of volatilomics

Studying olfaction is typically challenging since individual sensory experience varies [4] and is affected by biological, cultural, psychological, and environmental factors [16, 33, 71]. Historical medical traditions, including Ayurvedic and Chinese systems, emphasized sensory qualities such as aroma and taste as indicators of plant potency, reflecting experiential modes of knowledge rather than chemical concepts [38, 40, 54]. Nevertheless, some aspects of smell perception like odor intensity and pleasantness can be predicted through the physicochemical properties of odorants or VOCs [4, 47]. Of the 29 articles reviewed, 52% related informant’s perception to compounds by drawing on literature and online flavor databases, 45% recommended future phytochemical analysis, and only 10% performed phytochemical analysis to correlate the presence of compounds with informant’s perceptions (Supplementary Table S1). In a study focusing primarily on taste, local perceptions of tea bitterness correlated with total catechin content (associated with health-related properties of tea), but not with total methylxanthine content (associated with stimulating properties of tea) [2], suggesting that compounds beyond the pharmacologically active constituents may shape sensory perception. This aligns with recommendation in some studies included in this review to target compounds responsible for organoleptic properties, rather than focusing solely on dominant or pharmacologically active compounds [3]. For instance, low molecular weight VOCs in agarwood, which contribute to its characteristic aroma, were also found to exert the agarwood’s sedative effect in animal models [15]. Similarly, although VOCs in fruits account for only ∼0.01% of the fruit’s weight, minor compositional changes can drastically alter aroma profiles [1]; and differences between varieties of *Cannabis sativa* L. have been attributed to minor non-terpenoid VOCs rather than the major terpenes [68]. These findings highlight that sensory perception is often shaped by complex mixtures of compounds, rather than single dominant constituents. Such insights have been enabled by approaches that couple sensory analyses with volatilomics. Volatilomics is the qualitative and quantitative study of VOC mixtures emitted by living organisms [13]. Extraction techniques such as headspace sampling are employed to capture the VOCs emitted by plants followed by analysis using gas chromatography paired with flame ionization detector, mass spectrometer, or olfactometer (Fig. 4C). Studies that used modern technology (e.g., E-tongue) to be more objective in evaluating the influence of perception in plant selection also rely on compounds that will respond to sensors to record the taste [39, 45]. These studies claim that fingerprinting of the chemosensory nature of plants is possible and most of the time relate compound group to a sensory attribute, not a specific single compound [40]. However, aroma may not always be stable even within species as plants could exhibit intraspecific variation in specialized metabolites [46]. This variation, in turn, may influence how plant aromas are perceived and categorized, highlighting the importance of considering both chemical diversity and local sensory perception. Linking plant aroma chemistry with sensory descriptors and culturally embedded knowledge can contribute to a more comprehensive understanding of how aroma and olfaction inform the selection and utilization of plants.

## Conclusions

In summary, here we explored the influence of aroma on plant selection by examining how it is perceived, interpreted, and linked to plant use across disciplines through a structured scoping review. On the one hand, we find significant progress during the last decade in understanding the role of aroma in the selection and utilization of plants, with a transition from descriptive to quantitative studies. Across the reviewed literature, aroma is consistently described as contributing to plant selection within Indigenous and local knowledge systems, particularly through preliminary identification, therapeutic classification, and intergenerational knowledge transmission, though the evidence to date remains largely descriptive and associative rather than experimentally established. Our synthesis should therefore be interpreted as identifying patterns and gaps rather than establishing definitive causal relationships between aroma and plant selection.

On the other hand, there is still room for significant progress on three fronts. First, more unified approaches in data collection and analyses are needed (e.g., field protocols, type of data, greater language and ecological diversity). Studies typically differ in objectives and methods, which often limits direct comparability of results. Future studies could be more explicit about desired outcomes and strike a balance among the key focal elements—namely, plant knowledge, human perception, language, and plant chemistry. Second, the floristic context remains poorly understood. Lists of studied plants are often missing, and, to our knowledge, no study has systematically compared the aromatic plants selected by the communities against the broader regional species-pool they are exposed to; only two studies have tentatively explored the surrounding non-medicinal flora. Such comparisons are, however, essential to test for the (unique) organoleptic properties attributed to medicinal plants. Third, the integration of emic concepts with phytochemical analyses is still in its infancy. A better understanding of the role of aroma in human plant selection will require progress on all three fronts in combination to overcome the existing interdisciplinary divide.

## Supplementary Information

Below is the link to the electronic supplementary material.

Supplementary Material 1.

## Data Availability

All data are available in the source publications, and are included in this published article and its supplementary information files.

## Abbreviations

PRISMA: Preferred Reporting Items for Systematic reviews and Meta-Analyses
VOC: Volatile organic compound
VOS: Visualization of similarities
