# Supplementary material for: Does aroma influence Indigenous and local people’s selection and utilization of plants? A scoping review
Source: J Ethnobiol Ethnomed. 2026 Jul 17;22:94. doi: 10.1186/s13002-026-00930-4 (PMC13670547; doi:10.1186/s13002-026-00930-4)
Supplement: Supplementary file 1 [file 13002_2026_930_MOESM1_ESM.docx]

**Figure S1** PRISMA flow diagram illustrating the systematic review process.

**Table S1** The 29 references included in the present review. Listed according to publication year.

| Publication Year | Title | Author/s | Region: Culture | Information Source | Study Focus | Wild or cultivated plant/s | No. of species reported | Performed sensory studies? | Studied local odor language? | Related sensation to phytochemistry? | Performed phytochemical analyses? | Recommended phytochemical analyses? |
| --- | --- | --- | --- | --- | --- | --- | --- | --- | --- | --- | --- | --- |
| 1993 | Healing scents of the Andes | Howes & Classen | Neotropics: Inca | healers | medicinal plants | not stated | NA | yes | yes | no | no | no |
| 1998 | Medicinal and food plants: Isthmus Sierra Zapotec criteria for selection | Frei et al. | Neotropics: Zapotec | healers, general population | medicinal and food plants | not stated | 17 | yes | yes | no | no | yes |
| 1998 | Indigenous concepts of medicinal plants in Oaxaca, Mexico: Lowland Mixe plant classification based on organoleptic characteristics | Heinrich | Neotropics: Mixe | healers, general population | medicinal plants | cultivated | 213 | yes | yes | yes | no | yes |
| 1998 | Concepts of medicinal plants among the Nahua of the Sierra de Zongolica, Veracruz (Mexico) | Weimann & Heinrich | Neotropics: Nahua | healers | medicinal and food plants | not stated | NA | yes | yes | no | no | yes |
| 1999 | Yucatec Maya medicinal plants versus nonmedicinal plants: Indigenous characterization and selection | Ankli et al. | Neotropics: Yucatec Maya | healers | medicinal vs nonmedicinal plants | not stated | 398 | yes | yes | yes | no | yes |
| 2001 | Medicinal and ethnoveterinary remedies of hunters in Trinidad | Lans et al. | Neotropics: Indo-/Afro-Trinidadian | hunters | veterinary medicines | not stated | 40 | no | no | no | no | yes |
| 2002 | Medicinal plants of the Popoluca, México: organoleptic properties as indigenous selection criteria | Leonti et al. | Neotropics: Popoluca | healers | medicinal vs nonmedicinal plants | not stated | 150 | yes | yes | yes | no | no |
| 2004 | A sensory ecology of medicinal plant therapy in two Amazonian societies | Shepard | Neotropics: Matsigenka and Yora | general population | medicinal plants and illness | not stated | 537 | yes | yes | no | no | no |
| 2004 | Heterogeneity in Ethnoecological Knowledge and Management of Medicinal Plants in the Himalayas of Nepal: Implications for Conservation | Ghimire et al. | Palearctic: Indo-Aryans and Tibeto-Burmans | experts, local users | *Nardostachys* and *Neopicrorhiza* | not stated | 2 | yes | no | yes | no | no |
| 2007 | "Mints", smells and traditional uses in Thessaloniki (Greece) and other Mediterranean countries | Karousou et al. | Palearctic: Mediterranean | not specified | mints (*Acinos* and *Mentha*) | not stated | 8 | yes | no | yes | yes | yes |
| 2007 | Does the taste matter? Taste and medicinal perceptions associated with five selected herbal drugs among three ethnic groups in West Yorkshire, Northern England | Pieroni & Torry | Palearctic: Gujarati, Kashmiri, and English ethnic groups | general population | medicinal plants | not stated | 5 | yes (taste) | no | no | no | no |
| 2007 | Medicinal perceptions of vegetables traditionally consumed by South-Asian migrants living in Bradford, Northern England | Pieroni et al. | Palearctic: South Asian migrants | general population | vegetables | not stated | 25 | yes (taste) | no | no | no | no |
| 2008 | The importance of chemosensory clues in Aguaruna tree classification and identification | Jernigan | Neotropics: Aguaruna Jivaro | not specified | woody plants | not stated | 182 | yes | yes | no | no | no |
| 2009 | Chemosensory perception and medicinal plants for digestive ailments in a Mapuche community in NW Patagonia, Argentina | Molares & Ladio | Neotropics: Mapuche | general population | medicinal plants | both | 75 | yes | yes | no | no | no |
| 2014 | Can organoleptic properties explain the differential use of medicinal plants? Evidence from Northeastern Brazil | Medeiros et al. | Neotropics: sertaneja | family chiefs | medicinal plants | not stated | NA | yes | no | yes | no | no |
| 2014 | Medicinal plants in the cultural landscape of a Mapuche-Tehuelche community in arid Argentine Patagonia: an eco-sensorial approach | Molares & Ladio | Neotropics: Mapuche-Tehuelche | general population | medicinal plants | both | 121 | yes | no | no | no | no |
| 2015 | Taste of medicinal plants: A potential tool in predicting ethnopharmacological activities | Gilca & Barbulescu | Indomalayan: Indian | database | medicinal plants | not stated | 183 | yes (taste) | no | yes | no | yes |
| 2016 | Spectroscopic and E-tongue evaluation of medicinal plants: A taste of how rasa can be studied | Jayasundar & Ghatak | Indomalayan: Indian | e-tongue | medicinal plants | not stated | 7 | yes (taste) | no | yes | yes | yes |
| 2017 | The taste of heat: How humoral qualities act as a cultural filter for chemosensory properties guiding herbal medicine | Geck et al. | Neotropics: Zoque | healers | medicinal plants | not stated | 71 | yes | yes | yes | no | no |
| 2017 | An ethnobotanical investigation of traditional knowledge and uses of edible wild plants in the Umbria Region, Central Italy | Ranfa & Bodesmo | Palearctic: local people | general population | edible wild plants | not stated | 100 | yes (taste) | no | no | no | no |
| 2018 | Taste of phytocompounds: A better predictor for ethnopharmacological activities of medicinal plants than the phytochemical class? | Dragos & Gilca | Indomalayan: Indian | database | medicinal plants | not stated | 431 | yes (taste) | yes | yes | no | no |
| 2019 | Magic darts and messenger molecules: Toward a phytoethnography of indigenous Amazonia | Daly & Shepard | Neotropics: Makushi | not specified | medicinal plants | not stated | NA | yes (taste) | yes | yes | no | yes |
| 2021 | Wild plants and the food-medicine continuum—an ethnobotanical survey in Chapada Diamantina (Northeastern Brazil) | Medeiros et al. | Neotropics: specialists | experts | food and medicinal plants | wild | 46 | yes | no | no | no | no |
| 2021 | An ethnobotanical study on Indigenous food flavourings and aromatic enhancing plants used by the native communities of the central region of Sarawak | Yusli et al. | Indomalayan: local people | general population | plants used for flavourings, fragrance enhancers | both | 27 | yes | no | no | no | yes |
| 2022 | Taste and chemical composition as drives for utilitarian redundancy and equivalence: a case study in local medical systems in Northeastern Brazil | Reinaldo et al. | Neotropics: local people | experts | medicinal plants | not stated | 64 | yes (taste) | no | yes | no | yes |
| 2022 | No ambiguity: Chemosensory-based ayurvedic classification of medicinal plants can be fingerprinted using E-tongue coupled with multivariate statistical analysis | Jayasundar et al. | Indomalayan: Indian | e-tongue | medicinal plants | not stated | 78 | yes (taste) | no | yes | no | yes |
| 2023 | Untangling biocultural and socioeconomical drivers of African plum tree local nomenclature along a rural-urban gradient in central Cameroon | Lemoine et al. | Afrotropical: Beti | general population | *Dacryodes edulis* | cultivated | NA | yes (taste) | no | no | no | no |
| 2023 | A flavour perspective of Tiepishihu (Dendrobium officinale) - an emerging food ingredient from popular traditional Chinese medicinal plants: a review | Kirkwood et al. | Palearctic: Chinese | journal articles | *Dendrobium officinale* | both | NA | yes | no | yes | yes | yes |
| 2024 | Taste shaped the use of botanical drugs | Leonti et al. | Palearctic: local people | general population | botanical drugs in *De Materia Medica* | both | 407 | yes | no | yes | no | no |
